# Acidic Ionic Liquid as Both Solvent and Catalyst for Fast Chemical Esterification of Industrial Lignins: Performances and Regioselectivity

**DOI:** 10.3389/fchem.2019.00578

**Published:** 2019-08-14

**Authors:** Eric Husson, Lise Hulin, Caroline Hadad, Chaima Boughanmi, Tatjana Stevanovic, Catherine Sarazin

**Affiliations:** ^1^Unité de Génie Enzymatique et Cellulaire, UMR 7025 CNRS, Université de Picardie Jules Verne, Amiens, France; ^2^Laboratoire de Glycochimie, des Antimicrobiens et des Agroressources, UMR CNRS 7378, Université de Picardie Jules Verne, Amiens, France; ^3^Département des Sciences du Bois et de la Forêt, Centre de Recherche sur les Matériaux Renouvelables, Université Laval, Quebec City, QC, Canada

**Keywords:** lignin, chemical esterification, acidic ionic liquids, selectivity, thermal properties

## Abstract

Lignin can be considered an essential under-exploited polymer from lignocellulosic biomass representing a key for a profitable biorefinery. One method of lignin valorization could be the improvement of physico-chemical properties by esterification to enhance miscibility in apolar polyolefin matrices, thereby helping the production of bio-based composites. The present work describes for the first time a succeeded chemical esterification of industrial lignins with maleic anhydride in an acidic ionic liquid: 1-butyl-3-methyl imidazolium hydrogen sulfate without additional catalyst. This efficient strategy was applied to four industrial lignins: two softwood Kraft lignins (Indulin AT, Wayagamack), one hardwood Kraft lignin (Windsor), and one softwood organosolv lignin (Lignol), distinct in origin, extraction process and thus chemical structure. The chemical, structural, and thermal properties of modified lignins were characterized by ^31^P nuclear magnetic resonance, infrared spectroscopy and thermal analyses, then compared to those of unmodified lignins. After 4 h of reaction, between 30 to 52% of the constitutive hydroxyls were esterified depending on the type of lignin sample. The regioselectivity of the reaction was demonstrated to be preferentially orientated toward aliphatic hydroxyls for three out of four lignins (66.6, 65.5, and 83.6% for Indulin AT, Windsor and Lignol, respectively, vs. 51.7% for Wayagamack). The origin and the extraction process of the polymer would thus influence the efficiency and the regioselectivity of this reaction. Finally, we demonstrated that the covalent grafting of maleyl chain on lignins did not significantly affect thermal stability and increased significantly the solubility in polar and protic solvent probably due to additional exposed carboxylic groups resulted from mono-acylation independently of H/G/S ratio. Blending with polyolefins could then be considered in regard of compatibility with the obtained physico-chemical properties.

## Introduction

The biorefinery concept consists of the development of innovative and sustainable strategies for the valorization of a whole biomass such as a plant in its entirety and in particular the three main constitutive polymers; cellulose, hemicellulose and lignin, respectively (Ferreira, 2017). Lignins are complex highly branched amorphous polymers based on polyphenolic structures constituted of phenylpropane units, e.g., syringylpropane (S), guaiacylpropane (G), and hydroxyphenylpropane (H), providing interesting reactivity for chemical modifications (Erdtman, 1972; Stevanovic and Perrin, 2009; Laurichesse and Avérous, 2014). Lignin is henceforth considered as an essential under-exploited potential offering a key-issue for a profitable biorefinery (Calvo-Flores and Dobado, 2010; Doherty et al., 2011). Indeed, to date, only 2% of industrial lignin is valorized into applications other than energy production: base materials for the production of chemicals, adhesives or fertilizers are some examples (Gandini and Belgacem, 2008; Ion et al., 2018). An emergent way of valorization could be the blending of lignin with apolar matrices of polyolefins to produce partially bio-based composites with improved rheological and thermomechanical properties and better carbon footprint (Thielemans and Wool, 2005; Laurichesse and Avérous, 2014). However, the difference in polarity between lignins and polyolefins such as polyethylene impedes considerably their miscibility. To overcome this constraint, physico-chemical properties of lignin can be modified by chemical esterification with apolar moieties (Nadji et al., 2010; Gordobil et al., 2015). The acyl donors generally used for these modifications are short acyl chains present in acetic, butyric, succinic or maleic anhydrides (Xiao et al., 2001; Thielemans and Wool, 2005; Tamminen et al., 2012) or acyl chlorides (Koivu et al., 2016). Some drawbacks can be the use of tetrahydrofurane, 1,4-dioxane or *N*-methyl pyrrolidone as organic solvents, thionyl chloride as hazardous chemical reagents, and pyridine derivatives or 1-methyl imidazole as catalysts. Besides the use of non-environmentally friendly chemicals, drastic reaction conditions are often applied (high temperature reaction, extreme pH) together with the production of by-products and salts involving purification steps, which are not in agreement with the current environmental requirements and green chemistry framework (Anastas and Eghbali, 2010; Zhao et al., 2017). The development of alternative strategies thus remains a current scientific and technological challenge in the biorefinery concept. In this way, one can take advantage of the use of some ILs able to act both as solvent for lignin and as catalyst for lignin esterification based. For example, a recent study reported the IL to promote lignin acetylation of aliphatic hydroxyl groups while aromatic acetate were deacetylated, in DMSO as solvent (Suzuki et al., 2018). Earlier, based on the acidic properties of 1-butyl-3-methyl imidazolium hydrogen sulfate ([Bmim][HSO_4_]), it was evidenced that this IL acts as a catalyst for esterification of linear alcohols (Fraga-Dubreuil et al., 2002).

On the other hand, the access to a lignin fraction with an adequate purity or structural integrity for considering valorization requires an efficient fractioning of lignocellulosic biomass (LCB) upstream. From now on, a very large panel of fractioning, and delignification pretreatments of LCB is described in the literature. These include the use of dilute acid or alkali solutions, liquid hot water, organosolv, steam explosion, liquid hot water, ultrasounds-assisted processes, or high voltage electrical discharges methods (Park and Kim, 2012; Zhu et al., 2012; Putro et al., 2016; Brahim et al., 2017; Gominho et al., 2019). Some imidazolium-based ionic liquids (ILs) are now well-recognized for efficiently fractionate LCB under mild conditions (Brandt et al., 2011; Papa et al., 2012; Auxenfans et al., 2014; Husson et al., 2018; Singh et al., 2018). These ILs constitute promising solvents with unique properties such as low vapor pressure, recyclability, thermostability, and acceptable toxicity for some of them, particularly those with cation alkyl chain length inferior or equal to 4 carbons (García-Lorenzo et al., 2008; Egorova and Ananikov, 2014). In this context, it can be noticed that [Bmim] [HSO_4_] would be a suitable candidate to induce lignin removal from LCB as effective as acetate or chloride anion imidazolium-based IL. The acidic properties of this IL coupled with residual water content allow inducing acid-catalyzed hydrolysis of the β-O-4 linkage resulting in its dissociation from the carbohydrate matrix and then its dissolution (Brandt et al., 2011; Carvalho et al., 2015; Bernardo et al., 2019). It can then be imagined that chemical esterification of lignin could be directly implemented in the IL used for pretreatment/fractioning to avoid tremendous procedures of lignin extraction.

Before considering the development of a one-batch process including delignificaton of LCB and subsequent transformation of lignin in the same IL, the study of both the feasibility of chemical esterification of lignin in ([Bmim] [HSO_4_and the versatility of this strategy is inherent. For this reason, we selected four distinct industrials lignin as representative substrates: two softwood Kraft lignins (Indulin AT, Wayagamack), one hardwood Kraft lignin (Windsor) and one softwood organosolv lignin (Lignol) distinct in origin, extraction process, and thus chemical structure. The raw materials were firstly characterized, especially the hydroxyl groups. Then, the impact of single incubation in [Bmim][HSO_4_] on the structural and physicochemical properties was studied. Finally, the feasibility of lignin esterification with maleic anhydride was investigated without additional catalyst. Extracted modified lignins were finely characterized and the performances and selectivity of these non-conventional reaction systems were discussed based on quantitative data.

## Materials and Methods

### Reagent

Maleic anhydride (>99%), 2-chloro-4,4,5,5-tetramethyl-1,3,2-dioxaphospholane (95%), chromium acetylacetonate (>97%), *N*-Hydroxyphtalimide (97%), acetonitrile (HPLC grade), chloroform-d (99.8%), and pyridine (99.8%) were acquired from Sigma-Aldrich (Steinheim, Germany). 1-butyl-3-methylimidazolium hydrogen sulfate [Bmim][HSO_4_] (98%) was produced by Solvionic SA (Verniole, France).

### Industrial Lignins

Kruger Wayagamack and Domtar Windsor furnished two Kraft black liquors, used to precipitate Kraft lignin. Kruger Wayagamack black liquor was extracted from softwood and exhibited 50.9% of solid content with a pH = 14, and a volumetric mass of 1.27 g.ml^−1^. Domtar Windsor black liquor was from hardwood and contained 24.0% of solid with a pH = 13 and a volumetric mass of 1.12 g.ml^−1^. Wayagamack and Windsor lignins were extracted from black liquor followed by a precipitation procedure using carbon dioxyde described in a previous study (Schorr et al., 2014). Indulin AT lignin was extracted from softwood by Kraft process and furnished by the Westvaco Company. Composition in Klason and acid soluble lignins, total sugars and ash contents of these three Kraft lignins were reported by Schorr et al. (2014) and summarized in Supplementary Table 1 and suggested a satisfactory purity (>95% with around 1% of residual sugars) allowing to consider as significant the result on lignin esterification. Lignol™ lignin was extracted by organosolv process applied on softwood and produced by Lignol Innovation LTD (Berlin et al., 2011).

### Esterification of Industrial Lignins

The four industrial lignins were lyophilized and [Bmim][HSO_4_] was dried at 80°C under vacuum for 4 h (Rotavapor R-200, Büchi, France). Lignins and [Bmim][HSO_4_] were then stored in desiccator for 48 h before each reaction. The water content and water activity of the industrial lignins and [Bmim][HSO_4_] were then determined by Karl Fischer coulometry method (831 KF Coulometer, Metrohm, France) and a thermoconstanter (LabTouch Aw, Novasina, Switzerland), respectively. Data were reported in Table 1 as a mean of two replicates. Reactions were carried out in parallel with a synthesis station (Carousel 12+, Interchim, France) allowing to implement simultaneously 12 identical reactions. In a typical reaction, 300 mg of [Bmim][HSO_4_] (melting point 28°C) were incubated at 75°C under vigorous stirring until total liquefaction. Reaction was initiated by introducing lignin (10 mg) and maleic anhydride in distinct ratio: 1/1, 1/2, 1/5, 1/7.5, and 1/10 (w/w) and was performed at 75°C for 4 h under vigorous stirring. After this duration, the mixture was cooled down in an ice bath in order to stop the reaction. Acetonitrile was added in the reaction medium to precipitate the modified lignin. The resulted solid residue was collected by vacuum filtration, thoroughly washed with acetonitrile to eliminate unreacted maleic anhydride and [Bmim][HSO_4_], allowing to check that there is no residual IL, and then dried for 4 h at 100°C. Control experiments without maleic anhydride were also carried out in [Bmim][HSO_4_] (30/1 IL/lignin ratio, w/w) under the same conditions followed by similar extraction procedure to evaluate the impact of [Bmim][HSO_4_] on the four industrial lignins. Control, reaction and extraction were replicated three times.

**Table 1:** Water content and water activity of lyophilized lignins and dried [Bmim][HSO_4_].

	**Water content (%, w/w)**	**a_**w**_**
**Lignins**		
Indulin AT	3.24 ± 0.04	0.32
Windsor	3.12 ± 0.88	0.38
Wayagamack	3.23 ± 1.14	0.33
Lignol™	2.04 ± 0.70	0.34
**Ionic Liquid**		
[Bmim][HSO_4_]	0.02 ± 0.01	0.13

### Solubility of Lignins and Their Corresponding Esters

The solubility of Wayagamack and Windsor lignins (distinct by their respective H/G/S ratio) before and after esterification was determined in methanol and chloroform according to experimental procedure reported in previous studies (Cybulska et al., 2012; Sameni et al., 2017). At room temperature, 5 mL of organic solvent were added to 50 mg oven-dried lignin (or maleated lignin). The samples were sonicated for 10 min in a water bath sonicator at 40°C. The resulted suspensions were filtered with paper filter (Whatman no 1). Filters and retentats were air dried and then weighed. The soluble fraction was calculated by subtracting the insoluble fraction from the initial 50 mg lignin weight. Solubility values were expressed as mean values of three replicates with standard deviations (±) in g.L^−1^.

### Structural and Thermal Analyses

#### Pyrolysis-GC/MS

Pyrolysis-GC/MS analyses were performed in triplicate according experimental procedure described by Schorr et al. (2014). Each peak of chromatograms was identified according to NIST Mass Spectral Library and literature data (Meier and Faix, 1992). H/G/S ratio was calculated based on relative area (%) of each degradation product. All results were presented in Supplementary Table 2.

#### Infrared Spectroscopy

Raw, control and modified lignins were characterized by infrared spectrometry using a FTIR-8400S (Shimadzu, France) equipped with a universal ATR sampling accessory with diamond crystal. Solid samples, without further preparation, were analyzed between 4,000 and 600 cm^−1^ using 128 scans with a resolution of 4 cm^−1^. All spectra were normalized at 1,510 cm^−1^, the band assigned to aromatic rings vibration (Faix, 1991, 1992).

#### ^31^P NMR Analyses

Based on reference works from Argyropoulos et al., the quantification of lignin hydroxyls groups were performed by ^31^P NMR analyses (Argyropoulos, 1994, 1995). The sample preparation consisted in the derivatization of lignin hydroxyls with the phosphorylating agent: 2-chloro-tetra-1,3,2-dioxophospholane (TMDP). For this, 15 mg of dried lignin or modified lignin were introduced in a glass vial of 1.5 mL. 350 μL of pyridine/deuterated chloroform mixture (1.6/1 v/v) were then added followed by 100 μL of TMDP and 200 μL of a solution containing 55 mM of *N*-hydryphtalimide (internal standard) and 7.15 mM of chromium acetylacetonate solubilized in pyridine/deuterated chloroform mixture (1.6/1 v/v). Once prepared, the sample was immediately transferred in NMR tube. In this way, TMDP reacts with hydroxyl groups of lignin to generate phosphite derivatives, distinguishing aliphatic, phenolic hydroxyls, and carboxylic acids groups by their distinct chemical shift. ^31^P NMR spectra were acquired on a Bruker Avance III HD 500 MHz spectrometer equipped with BBI 5 mm probe operating at 202,4360 MHz (500,0800 MHz for ^1^H canal). Spectra acquisition, adapted from literature data (Crestini and Argyropoulos, 1997) was obtained by reverse pulse angle decoupling at 30°. Spectra consisted of 62 scans with spectral width of 81.5 kHz collected with a relaxation delay of 25 s at 298.1 K. Treatments of spectra were performed on Brucker TopSpin 3.2 software. The residual ^31^P signal of product issued from the reaction of water with the phosphorylating agent at 132.2 ppm was used as reference. Assignment was established according to literature data and gathered in Table 2 (Crestini and Argyropoulos, 1997; Crestini et al., 1998; Pu et al., 2011; Fiţigǎu et al., 2013). After integration, esterification yield (Y%) and regioselectivity (R%) were determined according to the equations below:

1. Y(HO_total_) = [(HO_aliph_+ HO_ph_)_control lignin_ - (HO_aliph_+ HO_ph_) _maleated lignin_]/(HO_aliph_+ HO_ph_)_control lignin_ x 100
2. R(HO_aliph_) = [(HO_aliph_)_control lignin_ - (HO_aliph_) _maleated lignin_]/[((HO_aliph_)_control lignin_ - (HO_aliph_) _maleated lignin_) + ((HO_ph_)_control lignin_ - (HO_ph_) _maleated lignin_)] x 100
3. R(HO_ph_) = [(HO_ph_)_control lignin_ - (HO_ph_)_lignin maleated_]/[((HO_aliph_)_control lignin_ - (HO_aliph_) _maleated lignin_) + ((HO_ph_)_control lignin_ - (HO_ph_) _maleated lignin_)] x 100

where HO_aliph_, HO_ph_ and HO_COOH_ correspond, respectively, to hydroxyls groups from aliphatic, phenolic and carboxylic acid moieties.

**Table 2:** Assignment of chemical shifts and integration regions in ^31^P NMR spectra of lignin after derivatization with 2-chloro-tetra-1,3,2-dioxophospholane adapted from literature data (Crestini and Argyropoulos, 1997; Crestini et al., 1998; Pu et al., 2011; Fiţigǎu et al., 2013).

**Structure**	**Abbreviation**	**Chemical shift δ (ppm)**
Aliphatic-OH	HO_aliph_	145.4–150.0
Aromatic-OH	HO_ph_	137.6–144.0
Syringyl-OH		~142.7
Guaiacyl-OH		139.0–140.2
p-hydroxyphenyl		~137.8
Carboxylic acid-OH	HO_COOH_	133.6–136.0

#### Thermal Gravimetric Analyses

Thermal Gravimetric Analyses (TGA) were performed on a Simultaneous Thermal Analyzer STA 449 C Jupiter Unit (Netzsch), at a heating rate of 10°C.min^−1^ under a constant argon flow of 50 mL.min^−1^ and from room temperature to 800°C (approximately 20 mg of each compound). Values of isothermal drift and sensitivity are 0.6 μg h^−1^ and 0.1 μg, respectively. The TGA apparatus is coupled with a Quadrupole QMS 403 Aeolos mass spectrometer, MS (Detector SEV/Sekundär Elektronen Vervielfacher (Channeltron), stainless steel capillary, counting time 20 ms per m/z with a resting time of 1 s, scanning width 1/51 amu).

#### Differential Scanning Calorimetry

Differential scanning calorimetric analyses (DSC) were carried out on a Netzsch DSC 204 F1 heat flux differential calorimeter. A constant heating rate of 10°C.min^−1^ was selected for analyses, from room temperature up to 160°C (1st heating), then cooling down to room temperature and finally heated to 200°C (2nd heating), under a constant argon flow of 200 mL.min^−1^ (approximately 6.0 mg of each compound).

## Result and Discussion

Chemical esterification of four industrial lignins were investigated in [Bmim][HSO_4_]. The catalytic properties of this IL (Gupta et al., 2007) associated to its β parameter of Kamlett Taft superior to 0.5 (Ventura et al., 2012) suggested that this non-conventional solvent could be a suitable alternative for our strategy in joining both lignin solvation and catalytic activity. However, before considering chemical modification of lignin, the structure of the starting lignins and the impact of incubation in [Bmim][HSO_4_] were assessed.

### Characterization of the Raw Lignins

As the potential of esterification depends on lignin hydroxyl groups and their accessibility/reactivity, we propose to first quantify each type of hydroxyl for all raw lignins and their respective H/G/S ratio by two methods: pyrolysis-GC/MS (Supplementary Table 2) and ^31^P NMR discussed below. Indulin AT, Lignol, and Wayagamack lignins exhibited similar distribution of the three phenylpropan units: 93–96% of G units, 1–4% of H units, and 2–3% of S units. This similarity can be explained by their softwood origin while the Windsor lignin originated from hardwood exhibits a different H/G/S ratio of 1/32/67. In Table 3 the hydroxyl quantification from ^31^P NMR spectra is reported. Indulin AT exhibited a total hydroxyl concentration (5.58 mmol.g^−1^ of lignin) superior to those of all others lignins. Distribution between HO_Ph_ and HO_aliph_ gave a ratio of 0.89 in agreement with previous studies (Fiţigǎu et al., 2013). Wayagamack lignin exhibited HO_Ph_/HO_aliph_ ratio of 1.09 (with a total hydroxyl concentration of 2.79 mmol.g^−1^ of lignin). In comparison to Indulin AT, these lower hydroxyls contents determined for Wayagamack lignin could be due to condensation reactions. This could be in agreement with the difference of condensation index between these two lignins (Supplementary Table 3). The hydroxyl concentrations can also decrease by oxidation reactions occurring during the precipitation step of the recovery process and leading to the formation of additional carboxylic functions (Gierer, 1985; Asgari and Argyropoulos, 1998; Kouisni, 2011). This could be correlated to the higher concentration of HO_COOH_ groups for Wayagamack in comparison to Indulin AT (0.64 mmol.g^−1^ of lignin for Wayaagmack vs. 0.40-0.48 mmol.g^−1^ of lignin for the three other). The Windsor lignin presented a total hydroxyl concentration of 3.87 mmol.g^−1^ of lignin with a HO_Ph_/HO_aliph_ ratio of 1.25. 60% of HO_Ph_ provided from S units. HO_Ph_ amount of Lignol lignin was similar to those from Indulin AT while the HO_aliph_ was lower. This lignin, issued from hardwood does not seem as sensitive to oxydation reaction as Wayagamack. Finally, Lignol lignin presented a hydroxyl group concentration of 3.23 mmol.g^−1^ of lignin with a HO_Ph_/HO_aliph_ ratio close to 1.Among the three lignins extracted from softwood, Lignol showed a lower condensed HO_Ph_ content, in agreement with the mild conditions of organosolv process. As the ratios evaluated by both methods are in close agreement, a classification of the four lignins according to their potential of esterification (concentration of hydroxyl groups susceptible to be implied in O-acylation reaction) can be suggested: Indulin AT > Windsor > Wayagamack ≈ Lignol.

**Table 3:** Quantification by ^31^P NMR of hydroxyl groups (mmol.g^−1^ of lignin) in raw lignins.

**Concentration (mmol.g^**−1**^ of lignin)**	**Industrial lignins**			
	**Indulin AT**	**Wayagamack**	**Lignol**	**Windsor**
HO_aliph_	2.19 ± 0.01	1.34 ± 0.07	1.39 ± 0.02	1.56 ± 0.12
HO_Ph_	1.95 ± 0.06	1.45 ± 0.28	1.37 ± 0.14	1.93 ± 0.01
HO_Syringyl_	nd	nd	nd	1.17 ± 0.06
HO_Guaiacyl_	1.43 ± 0.01	1.17 ± 0.01	1.14 ± 0.11	0.56 ± 0.04
HO_Hydroxyphenyl_	0.08 ± 0.01	0.03 ± 0.01	0.05 ± 0.02	0.04 ± 0.01
Condensed HO_Ph_	0.44 ± 0.07	0.25 ± 0.04	0.19 ± 0.01	0.17 ± 0.10
HO_COOH_	0.44 ± 0.03	0.64 ± 0.01	0.48 ± 0.02	0.39 ± 0.02
${\text{HO}}_{\text{total}}^{\text{a}}$	4.58 ± 0.07	3.43 ± 0.05	3.23 ± 0.14	3.87 ± 0.14
${\text{HO}}_{\text{esterifiable}}^{\text{b}}$	4.14 ± 0.04	2.79 ± 0.04	2.75 ± 0.12	3.49 ± 0.13
Ratio HO_Ph_/HO_aliph_	0.89 ± 0.04	1.09 ± 0.08	0.99 ± 0.11	1.25 ± 0.09
Ratio H/G/S	4/96/0	2/98/0	3/97/0	2/38/60
Ratio H/G/S^c^	4/94/2	1/96/3	4/93/3	1/32/67

a *HO_total_ = HO_aliph_ + HO_Ph_ + HO_COOH_*.

b *HO_esterifiable_ = HO_aliph_ + HO_Ph_*.

c *Calculated using pyrolysis-GC-MS analyses*.

*nd, not detected*.

### Impact of Incubation in [Bmim][HSO_4_] on Lignin Properties in View of Further Transformation

#### Structural Properties

**Figure 1:**
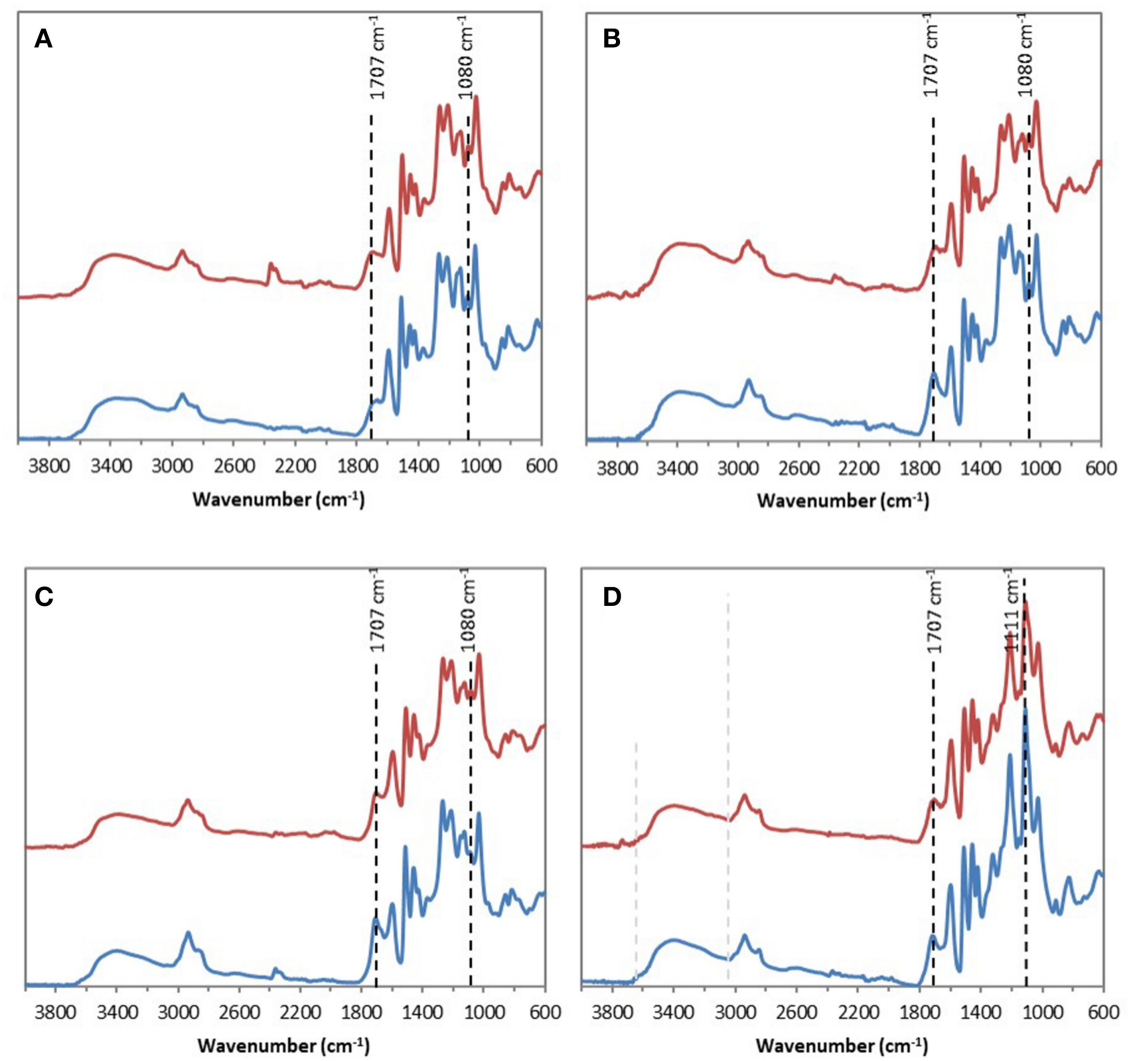
ATR–FTIR spectra of the four industrial lignins: Indulin AT **(A)**, Wayagamack **(B)**, Lignol **(C)**, and Windsor **(D)** before (blue) and after incubation in [Bmim][HSO_4_] for 4 h at 75°C (red).

**Figure 2:**
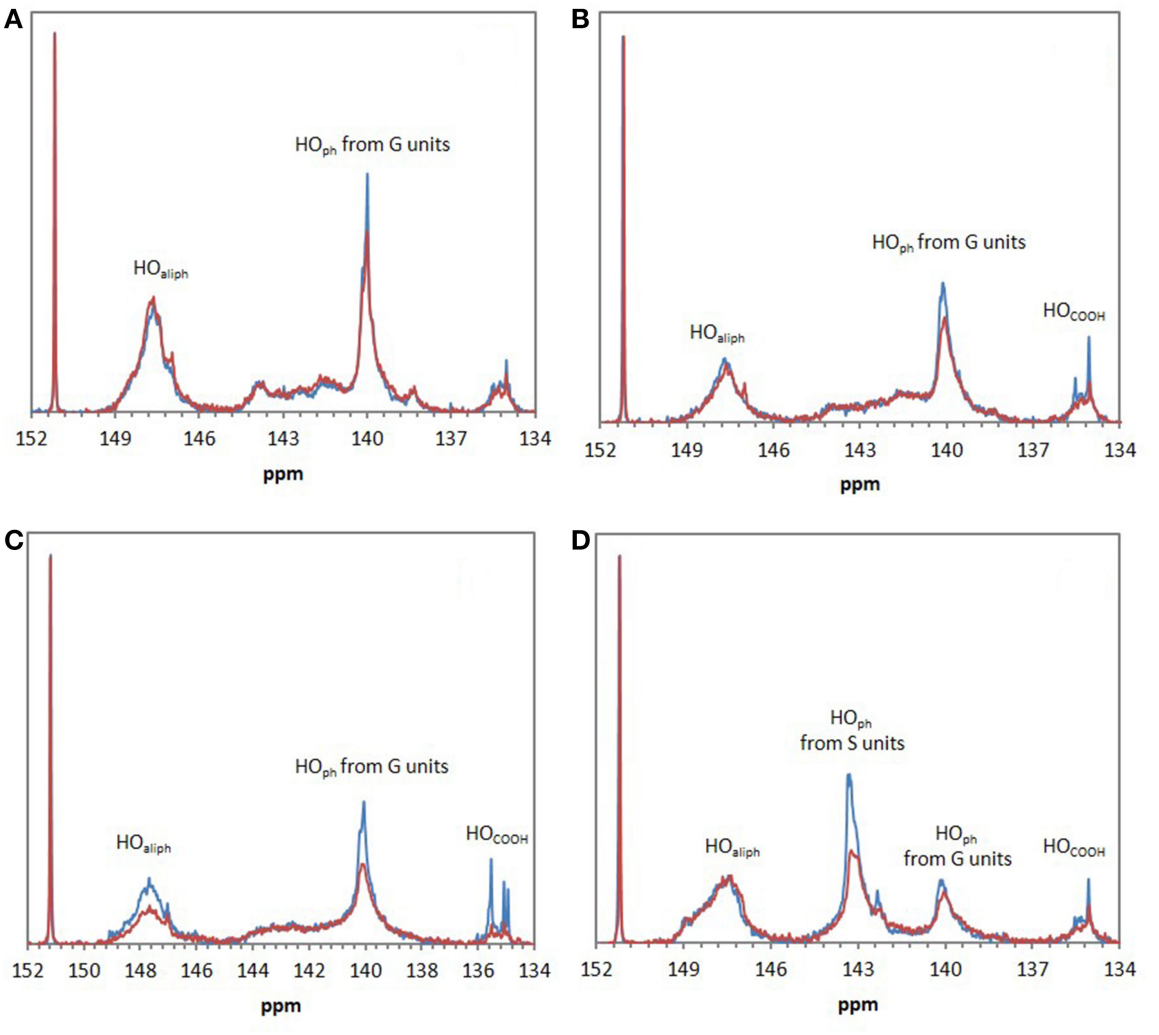
^31^P NMR spectra of the four phosphorylated industrial lignins: Indulin AT **(A)**, Wayagamack **(B)**, Lignol **(C)**, and Windsor **(D)** before (blue) and after incubation in [Bmim][HSO_4_] for 4 h at 75°C (red). *N*-hydroxyphtalimide was used as internal standard with chemical shifts of phosphitylated *N*-hydroxy *N*-hydroxyphtalimide centered on 152.2 ppm.

FTIR analyses of the four industrial lignins (Indulin AT, Wayagamack, Lignol, and Windsor) were performed after incubation in [Bmim][HSO4] for 4 h at 75°C, and extraction process. The obtained data were compared to the corresponding raw materials (Figure 1). FTIR spectra of Indulin AT (Figure 1A) before and after incubation in [Bmim][HSO_4_] did not evidence significant differences suggesting the preservation of overall structural integrity of this lignin in these conditions, as already suggested in the literature for other imidazolium-based ILs (Hulin et al., 2015). This observation is in agreement with the similar quantification of hydroxyl group of Indulin AT before (Table 3) and after incubation in the IL (Table 4). For Wayagamack, Lignol and to a lesser extent Windsor lignins (Figures 1B–D), a slight decrease in intensity of the band at 1,707 cm^−1^, characteristic of carbonyl stretching, was observed. This could be due to reaction implying ketone functions catalyzed by [Bmim][HSO4] (Gupta et al., 2007). It was also noticed that for the Wayagamack and Lignol lignins a slight increase in the band intensity at 1,080 cm^−1^ occurred, assigned to the C-O deformation in aliphatic esters and secondary hydroxyls (Casas et al., 2012). In this way, aldolization and/or ketolization catalyzed by [HSO_4_] anion may occur between lignin ketones and electrophile groups, leading to the formation of C-C covalent bonds and secondary alcohols. This aldolization can also be suggested for the Windsor lignin by the diminution of the band intensity at 1,111 cm^−1^, assigned to C-O of aliphatic ethers. Anyhow, these modifications on FTIR spectra, revealed after incubation in [Bmim][HSO_4_] of raw lignins, remained minor. Additional information was provided by ^31^P NMR spectra on the distinct types of hydroxyl groups than FTIR cannot discriminate (Pu et al., 2011). Figure 2 shows ^31^P NMR spectra of the four phosphorylated industrial lignins (Indulin AT, Wayagamack, Windsor and Lignol) before and after incubation in [Bmim][HSO_4_]. Decreases of peak intensity of phenolic hydroxyls from G units (HO_ph_, 139.0–140.2 ppm) and carboxylic acid hydroxyls (HO_COOH_, 133.6–136.0 ppm) were observed for Wayagamack, Windsor, and Lignol lignins (Figures 2B–D). In the case of Indulin AT, the intensity decrease was much less marked (Figure 2A). Contrary to other lignins, intensity of HO_aliph_ peak from Lignol decreased significantly (Figure 2C). Concerning the Windsor, intensity of peaks assigned to HO_ph_ from the S units was more strongly decreased than those from G units after incubation in [Bmim][HSO_4_] (Figure 2D). Although the overall structural integrity of lignins would be preserved after incubation in [Bmim][HSO_4_], some chemical modifications specifically affecting amount aliphatic, phenolic, and carboxylic acid hydroxyl groups occurred. These observations are reinforced by quantifications reported before incubation in IL of raw lignins (Table 3) and after incubation (Table 4). This could be due to condensation reactions between β-ketone and hydroxyl or β-carbonyl groups in acidic conditions (Wayman and Lora, 1980; Hussin et al., 2014). In addition, decrease in HO_ph_ may be due to dehydration resulting from acid-catalyzed elimination reactions (El Hage et al., 2009; Hussin et al., 2014).

**Table 4:** Quantification by ^31^P NMR of hydroxyl groups concentration (mmol.g^−1^ of lignin) in control lignin (incubated in [Bmim][HSO_4_] for 4 h at 75°C without maleic anhydride) and in maleated lignins and regioselectivity of the reaction (R%).

	**Control lignin (mmol.g**^****−1****^ **of lignin)**		**Lignin maleate (mmol.g**^****−1****^ **of lignin)**		**Regioselectivity (%)**	
	**HO_**aliph**_**	**HO_**ph**_**	**HO_**aliph**_**	**HO_**ph**_**	**HO_**aliph**_**	**HO_**ph**_**
Indulin AT	2.29 ± 0.17	1.66 ± 0.16	0.88 ± 0.47	1.00 ± 0.36	66.6 ± 3.7	33.4 ± 3.7
Wayagamack	0.79 ± 0.38	1.10 ± 0.07	0.31 ± 0.02	0.77 ± 0.08	51.7 ± 11.7	48.3 ± 11.7
Lignol	1.18 ± 0.29	1.20 ± 0.04	0.50 ± 0.10	1.07 ± 0.01	83.6 ± 0.9	16.4 ± 0.9
Windsor	1.26 ± 0.42	1.43 ± 0.16	0.55 ± 0.01	1.00 ± 0.20	65.5 ± 4.9	34.5 ± 4.9

#### Thermal Properties

The thermal properties of the four industrial lignins were characterized by thermogravimetric analysis (TGA) before (Supplementary Figure 1) and after (Supplementary Figure 2) incubation in [Bmim][HSO_4_]. TGA curves represent weight loss of lignin relative to temperature of thermal degradation. The first derivative of the corresponding curve (DTG) shows rate of weight loss. The peaks of the DTG curves may be defined as thermal degradation temperatures: T_onset_ as the temperature at which the degradation of the polymer starts, T_50%_ as the temperature at which lignin sample attained 50% of degradation and DTG as thermal decomposition temperature at which maximal decomposition occurs. Table 5 summarizes thermal analyses data relative to the four lignins before and after incubation in [Bmim][HSO_4_]. TGA thermograms suggested that thermal degradation behavior was different after incubation in IL (Supplementary Figures 1, 2). The incubation seemed to minimize the differences between T_onset_ value of each lignin. Indeed, these values ranged between 200.0 and 215.7°C after incubation vs. 145.0 and 183.3°C for raw materials. After incubation in IL, thermal degradation of the four lignins was slower and required higher temperature for starting. However, IndulinAT exhibited a decrease in T_50%_ and relative mass loss at 798°C after incubation in [Bmim][HSO_4_]. The increase of T_onset_ after incubation could be related to the decrease in carboxylic acid hydroxyl groups evidenced by ^31^P NMR and FTIR spectra. This effect would be particularly marked for Lignol lignin. Previous results obtained by ^31^P NMR suggested that incubation in [Bmim][HSO_4_] decreases the concentration in HO_ph_ of lignins_._ These HO_ph_ groups allow the prevention of autocondensation of lignin during thermal decomposition (Zhao et al., 2014). We suggested that IL incubation of Wayagamack, Lignol, and Windsor lignins would generate some more condensed aromatic structures leading to higher stability as revealed by increase in T_onset_. Glass transition temperature (Tg) of the raw lignins were then determined by DSC and ranged between 135.5 and 150.0°C. Although not precisely ascertainable in our experiments, there is an effect of IL incubation that could be due to variation in hydroxyl groups, or maybe the presence of low molecular weight contaminants or residual solvent (Vasile and Zaikov, 2006; Sadeghifar et al., 2012 Cui et al., 2013).

**Table 5:** Thermal analyses summary of lignins before and after incubation in [Bmim][HSO_4_] for 4 h at 75°C.

	**Lignins**	**T_**onset**_ (°C)**	**T_**50%**_ (°C)**	**Residual relative mass^a^ (%)**	**DTG max (°C)**
Raw	Indulin AT	145.0	645.7	46.6	349.0
	Wayagamack	183.3	516.0	41.3	397.3
	Lignol	162.5	461.2	38.5	390.9
	Windsor	165.0	482.8	39.7	359.9
After incubation in [Bmim][HSO_4_]	Indulin AT	215.7	572.3	44.0	338.4
	Wayagamack	211.3	552.0	43.6	338.8
	Lignol	200.0	631.6	46.9	335.5
	Windsor	208.8	632.9	46.8	342.4

a *Residual relative mass (%) determined at 798°C*.

All together, these characterizations provided evidence that control lignins obtained after IL incubation of raw lignins left a good potential in hydroxyl groups for esterification.

### Chemical Esterification of Lignins in [Bmim][HSO_4_]

#### Determination of Suitable Lignin/Maleic Anhydride Ratio

**Figure 3:**
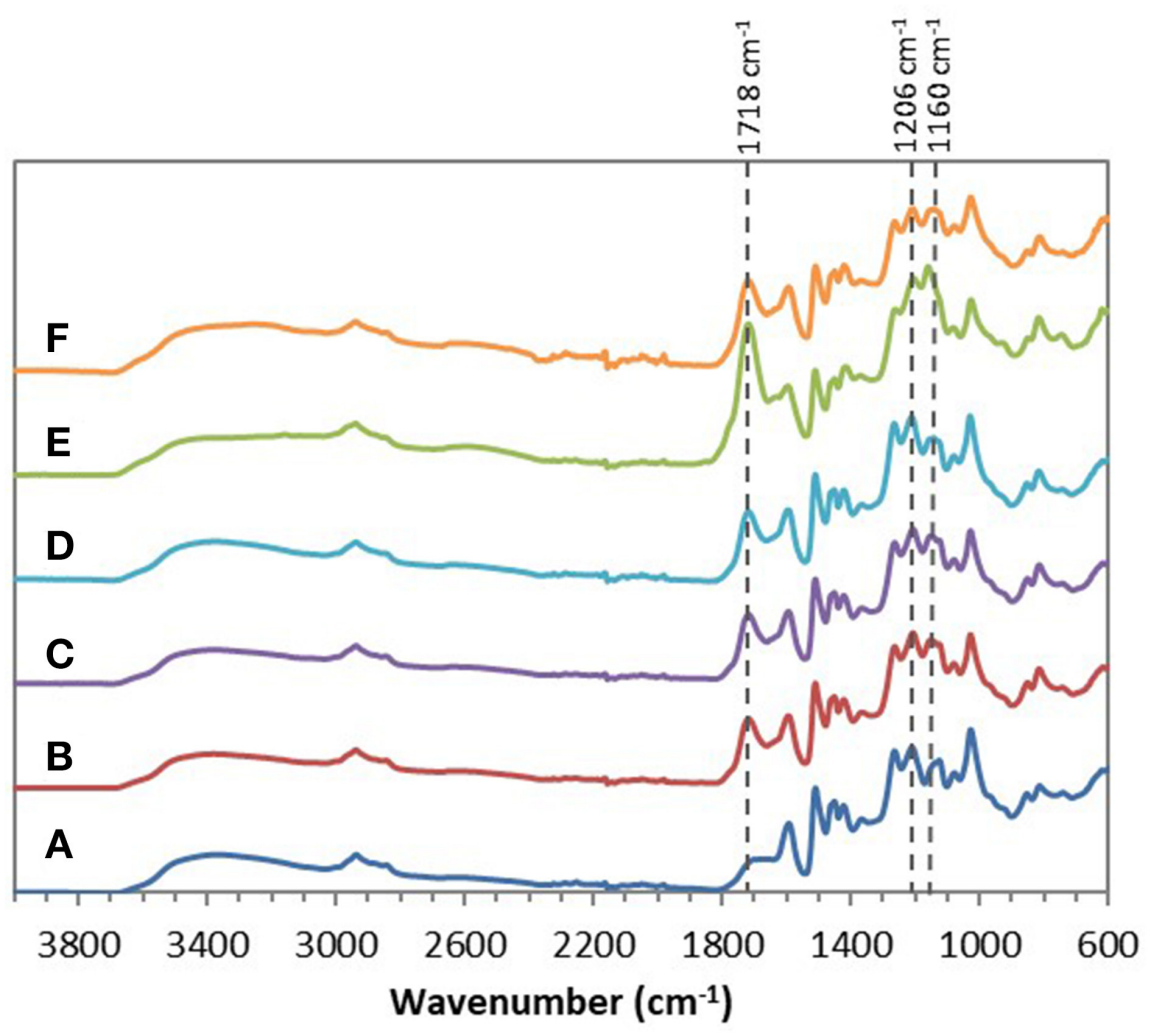
ATR–FTIR spectra of Indulin AT after incubation in [Bmim][HSO_4_] for 4 h at 75°C **(A)** and after esterification in similar conditions with the lignin/maleic anhydride ratio of 1/1 w/w **(B)**, 1/2 w/w **(C)**, 1/5 w/w **(D)**, 1/7.5 w/w **(E)**, and 1/10 w/w **(F)**.

**Figure 4:**
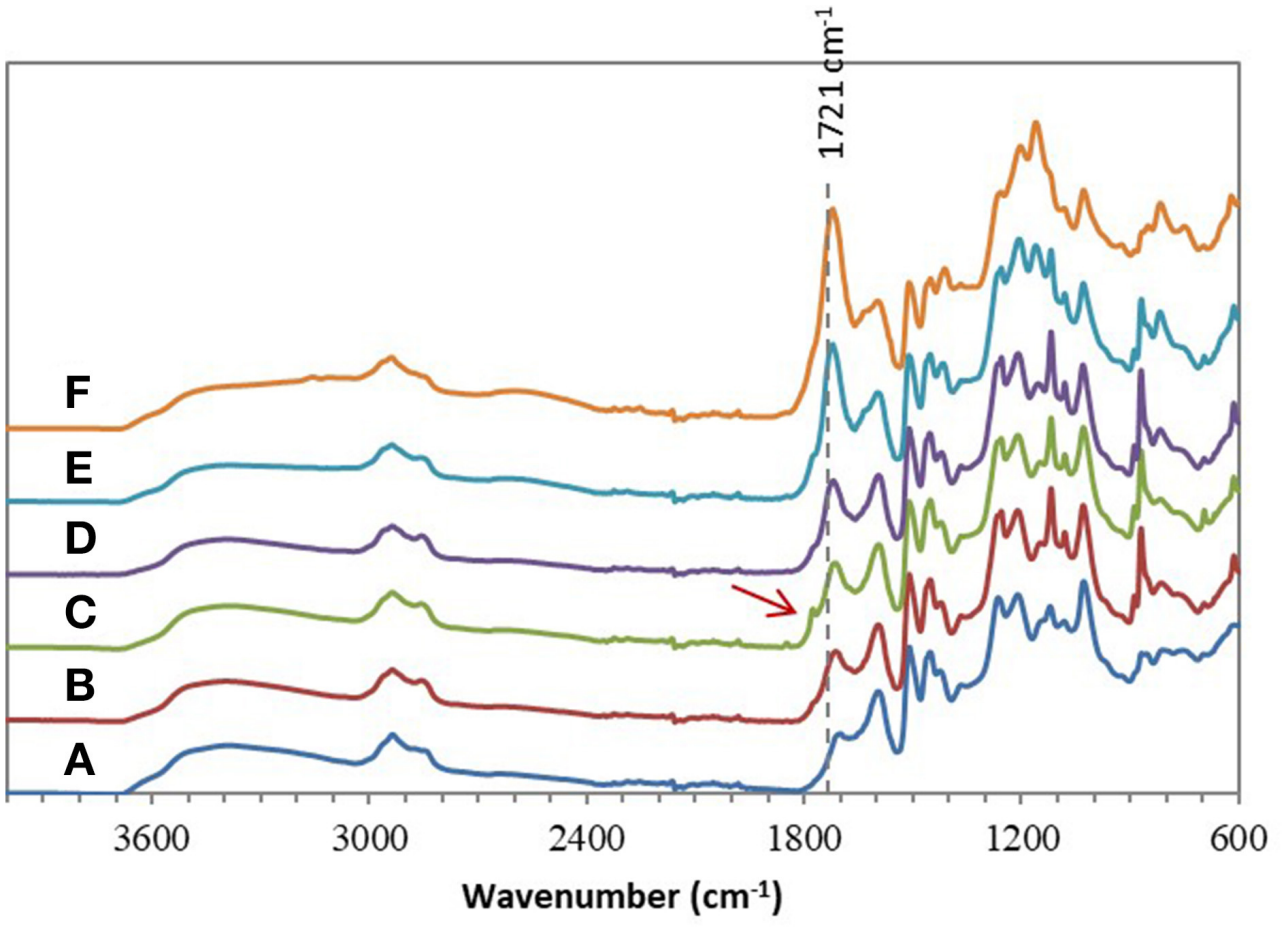
ATR–FTIR spectra of Lignol after incubation in [Bmim][HSO_4_] for 4 h at 75°C **(A)** and after esterification in similar conditions with the lignin/maleic anhydride ratio of 1/1 w/w **(B)**, 1/2 w/w **(C)**, 1/5 w/w **(D)**, 1/7.5 w/w **(E)**, and 1/10 w/w **(F)**.

**Figure 5:**
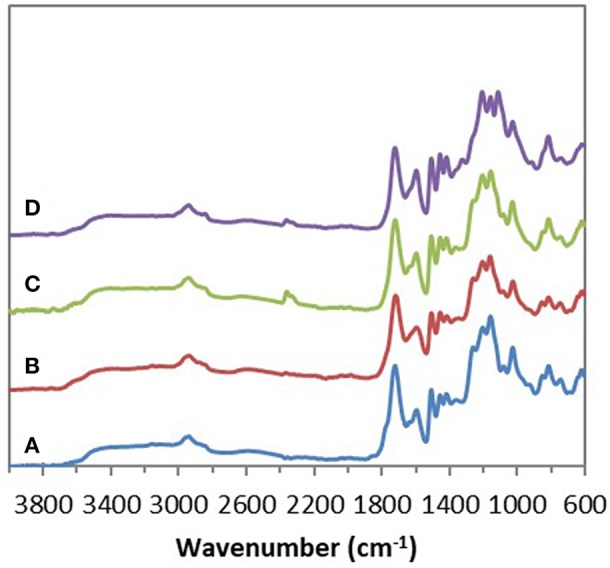
ATR–FTIR spectra of the four maleated lignins: Indulin AT **(A)**, Lignol **(B)**, Wayagamack **(C)**, and Windsor **(D)** after esterification in [Bmim][HSO_4_] for 4 h at 75°C with the lignin/maleic anhydride ratio of 1/7.5 w/w.

Chemical esterifications of Indulin AT and Lignol, two industrial softwood lignins distinct by their respective extraction processes (Kraft vs. organosolv), were firstly performed in [Bmim][HSO_4_] with various lignin/maleic anhydride ratio (w/w) to target the optimal conditions. For this study, a temperature of 75°C and a duration of 4 h were selected based on a previous work concerning chemical esterification of lignin in dioxane (Schorr et al., 2014). After incubation in [Bmim][HSO_4_] in presence of various maleic anhydride amount, all FTIR spectra of recovered Indulin AT (Figure 3) showed the presence of a characteristic band of ester carbonyl at 1,718 cm^−1^ not observed on the FTIR spectrum of incubated Indulin AT without acyl donor (Figure 1A). This carbonyl ester band is distinct from those of maleic anhydride (1,737 cm^−1^) and those assigned to free carboxylic group (1,707 cm^−1^). The presence of this band at 1,718 cm^−1^ was supported by significant increase of band intensities at 1,206 cm^−1^ and 1,160 cm^−1^, corresponding to C-C and C=O stretching and C=O from conjugated ester groups, respectively (Faix, 1992; Boeriu et al., 2004). These characteristic bands suggested the feasibility of the chemical esterification of Indulin AT lignin in [Bmim][HSO_4_]. Esterification in this IL would be more efficient with the lignin/maleic anhydride ratio of 1/7.5 w/w (Figure 3E and Figure 5A) while the best ratio in dioxane was 1/2 w/w (Schorr et al., 2014). To confirm the relevance of this lignin/maleic anhydride ratio, similar reactions were performed with Lignol lignin. The FTIR spectra of recovered Lignol were presented in Figure 4. From the ratio of 1/1 w/w, the band at 1,721 cm^−1^, characteristic of ester carbonyl, appeared. The intensity of this band increased as the higher ratio. A slight band at 1,176 cm^−1^ (see red arrow on Figure 4C) can be observed, maybe assigned to carbonyl from aromatic ester (Barra et al., 1999; Maldhure et al., 2012). The increase of band intensity at 1,124 cm^−1^, assigned to secondary alcohols and ester carbonyl groups, also confirmed the synthesis of lignin maleate (Faix, 1992; Casas et al., 2012). For 1/7.5 and 1/10 w/w ratio, the intensity of the band at 1,161 cm^−1^ increased and constituted thus a supplementary proof of chemical esterification of lignin (see spectra on Figures 4D–F and Figure 5B). Based on these results, chemical esterifications of the two other industrial lignins (Wayagamack and Windsor) with maleic anhydride were performed with the ratio lignin/maleic anhydride1/7.5 w/w. After extraction from reaction media, FTIR analyses of recovered lignins confirmed the feasibility of chemical esterification of Wayagamack, and Windsor lignins as illustrating by their corresponding infrared footprint (Figures 5C,D).

#### Reaction Performances and Selectivity

**Figure 6:**
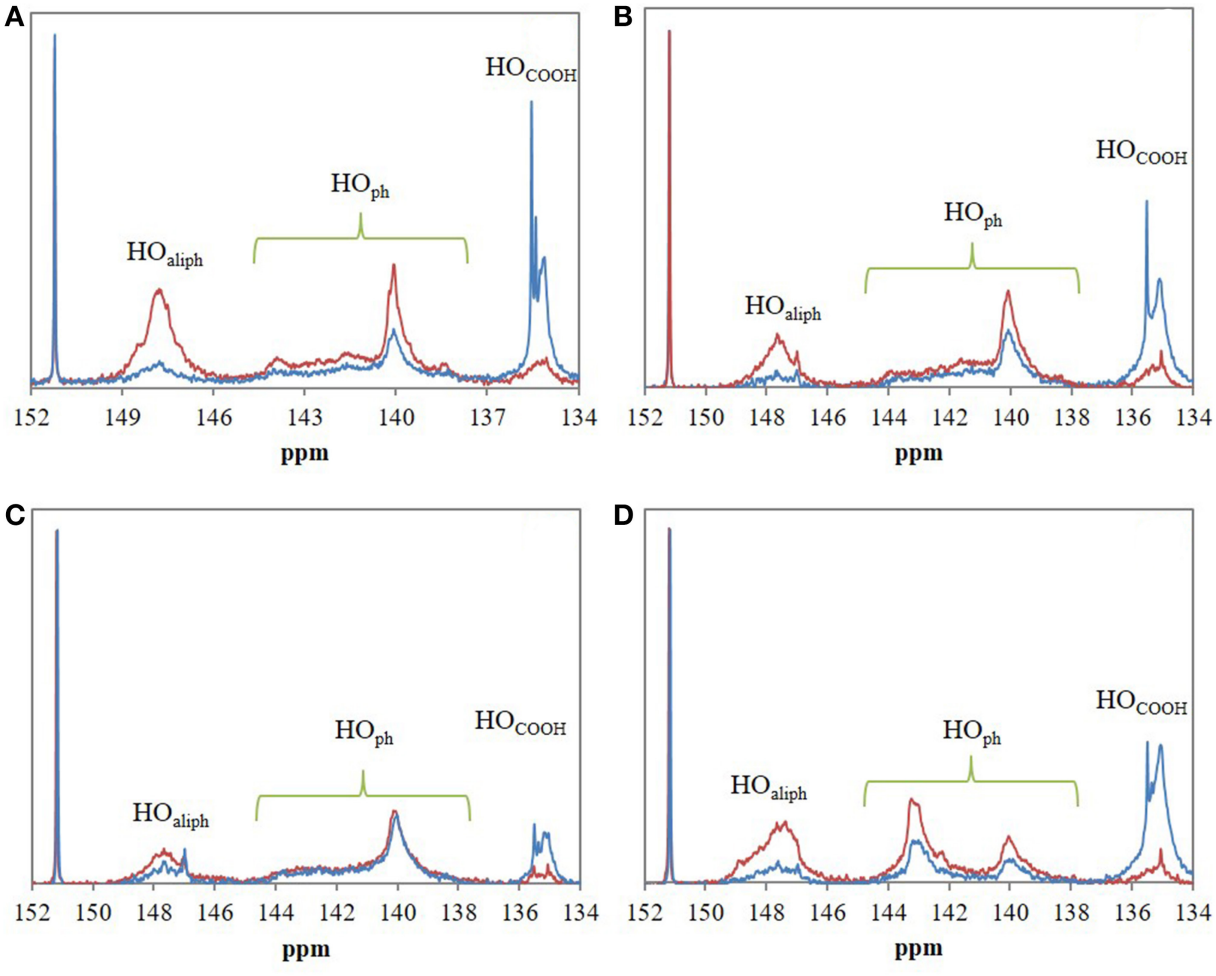
^31^P NMR spectra of the four industrial lignins: Indulin AT **(A)**, Wayagamack **(B)**, Lignol **(C)**, and Windsor **(D)** after incubation in [Bmim][HSO_4_] for 4 h at 75°C without maleic anhydride (control lignins, red spectra) and with maleic anhydride (maleated lignins, blue spectra). *N*-hydroxyphtalimide was used as internal standard with chemical shifts of phosphitylated *N*-hydroxy *N*-hydroxyphtalimide around 150.7–153.6 ppm. Esterification was performed with the lignin/maleic anhydride ratio of 1/7.5 w/w.

Figure 6 evidences significant differences between ^31^P NMR spectra of control lignins (incubated in [Bmim][HSO_4_] for 4 h at 75°C without maleic anhydride) and esterified lignins. In most of the case, ^31^P NMR spectra of esterified lignins present a decrease of peak intensity of HO_aliph_ (145.4–150.0 ppm) and HO_ph_ (139.0–140.2 ppm) as compared to control lignins spectra. A sharp increase of peak intensity corresponding to HO_COOH_ (133.6–136.0 ppm) can be noticed. The esterification would thus target both aliphatic and phenolic hydroxyls of lignins as already observed in FTIR spectra. Moreover, the increase of hydroxyls from carboxylic groups confirmed the presence of grafted maleyl chains on lignin by exposing free one-end carboxylic groups of anhydride. Quantitative analyses of ^31^P NMR spectra were used to determine esterification yield (Y in%) and then to deduce the regioselectivity of the reaction (Table 4). Global esterification yields obtained for Lignol and Wayagamack (30.1 and 38.5%, respectively) were significantly lower than those obtained with Windsor and Indulin AT (47.4 and 52.5%, respectively). The quantitative results thus suggested a difference of esterification performances between these four industrial lignins, maybe influenced either by the origin (softwood or hardwood) or the extraction process (Kraft or organosolv). The comparison of these performances with literature data is not an easy one. Indeed yields were often expressed as the mass increase of recovered lignin after esterification, unfortunately biased by mass loss during extraction from reaction medium and/or residual adsorption of acyl donor (Nadji et al., 2010; Cachet et al., 2014; Chatterjee et al., 2014; Gordobil et al., 2016). However, our obtained yields seemed to be competitive with some of those reported in literature. For instance, a mass increase of 3.3% was determined for esterification of the organosolv lignin with maleic anhydride in tetrahydrofuran. This yield corresponded to 0.5 mmol of esterified hydroxyl groups per gram of lignin (mmol/g_lignin_) (Chatterjee et al., 2014). This result was significantly lower than those obtained with our greener strategy for the four tested lignins: in average 1.20 mmol/g_lignin_ of esterified hydroxyls achieved in shorter duration.

Literature data usually suggests that esterification would mainly occur directly or indirectly on aliphatic hydroxyls (Thielemans and Wool, 2005; Nadji et al., 2009; Ahvazi et al., 2011; Maldhure et al., 2011, 2012; Chatterjee et al., 2014; Suzuki et al., 2018). In this context, we proposed to finely define the resulted regioselectivity from our reaction system for the four industrial lignins. It can be observed that HO_aliph_ were preferentially esterified for Indulin AT, Lignol, and Windsor lignins (Table 4). These results agreed with the low selectivity in favor to HO_ph_ from lignin suggested in a previous study (Ahvazi et al., 2011). Interestingly, similar part of HO_ph_ were esterified (around 33% of total esterified hydroxyl groups) for Indulin AT and Windsor: two lignins extracted from wood by Kraft process. This regioselectivity could be explained by the high amount of syringyl units with two methoxy groups on the aromatic ring inducing probably steric hindrance in the molecular environment of HO_ph_ (Thielemans and Wool, 2005). Concerning the Wayagamack lignin, hydroxyl groups were esterified regardless of their respective nature (51.7% of HO_aliph_ vs. 48.3% of HO_ph_). This absence of regioselectvity could suggest that chemical modification of lignin occurred during extraction process. About Lignol lignin, HO_aliph_ were preferentially esterified with a regioselectivity of 83.6%. Although organosolv process preserved the native structure of lignin and thus the amount in coniferyl alcohol, we expected a higher availability of HO_ph_ for esterification with maleic anhydride. By this way, we suggested that the solvation in [Bmim][HSO_4_] could affected the reactivity of HO_ph_ in agreement with results in dioxane previously reported in the literature (Ahvazi et al., 2011). In solution, maleic anhydride exhibits two carboxylic functions allowing either mono-acylation or di-acylation. The HO_COOH_/HO_esterified_ ratio can distinguish between these two possible reactions. For Lignol and Wayagamack lignins, the ratio was, respectively, of 0.94 and 0.92 suggesting a mechanism exclusively oriented toward mono-acylation. On the contrary, the ratio of 0.59 determined for Indulin AT, suggested that 1/3 of maleic anhydride induced di-acylation on lignin.

### Thermal Properties of Lignin Maleate

Table 6 compares thermal analyses data relative to the four lignins after esterification in [Bmim][HSO_4_] with maleic anhydride. TGA and DTG thermograms of the four lignins after esterification exhibited significant differences in comparison with control lignins (Supplementary Figure 3 vs. Supplementary Figure 2). Indeed, thermograms of the four maleated lignins presented henceforth two characteristic temperatures of maximal degradation (DTG1_max_ and DTG2_max_). DTG1_max_ ranged between 192.2 and 196.3°C (Table 6). This new DTG1_max_ could be due to the thermal decomposition of the covalently grafted maleyl chains on polymers. Overall, it can be noticed a decrease in thermal stability for the four maleated lignins as illustrated by the lower T_onset_ and T_50%_ (except for maleated Wayagamack lignin) than those determined for corresponding control (Table 2). About maleated Wayagamack, no significative change of T_50%_ was detected as already reported (Schorr et al., 2014). In addition, TGA-MS coupled analyses of lignin maleates evidenced the generation of degradation fragments with m/z of 26 (C_2_H_2_) and 44 (CO_2_). Example of fragments generated during TGA-MS coupled analysis of maleated Indulin AT lignin was presented in Supplementary Figure 4. These fragments would be assigned to thermal decomposition of maleic chains (Cascaval et al., 1996; Chen et al., 2013). Combined with ^31^P NMR analyses, these thermal degradation fragments strengthen the proof of concept of efficient lignin esterification in [Bmim][HSO_4_] with maleic anhydride without additional catalyst. Previous works about esterification of lignins demonstrated that Tg decreased drastically after efficient covalent grafting of alkyl chain on the polymer (Schorr et al., 2014; Hulin et al., 2015). This thermal behavior of modified lignins was thus consistent with a succeeded esterification.

**Table 6:** Thermal analyses summary of lignins after chemical esterification in [Bmim][HSO_4_] with maleic anhydride for 4 h at 75°C.

**Modified Lignins**	**T_**onset**_ (°C)**	**T_**50%**_ (°C)**	**Residual relative mass^a^ (%)**	**DTG_**1**_ max^b^ (°C)**	**DTG_**2**_ max^c^ (°C)**
Indulin AT	147.1	478.4	40.0	196.3	324.1
Wayagamack	172.4	557.9	43.8	196.8	318.2
Lignol	155.8	491.5	41.1	192.2	325.0
Windsor	160.0	499.2	40.7	193.5	336.9

a *Residual relative mass (%) determined at 798 °C*.

b *First maximum degradation temperature*.

c *Second maximum degradation temperature*.

### Solubility of Raw Lignins vs. Maleated Lignins

**Figure 7:**
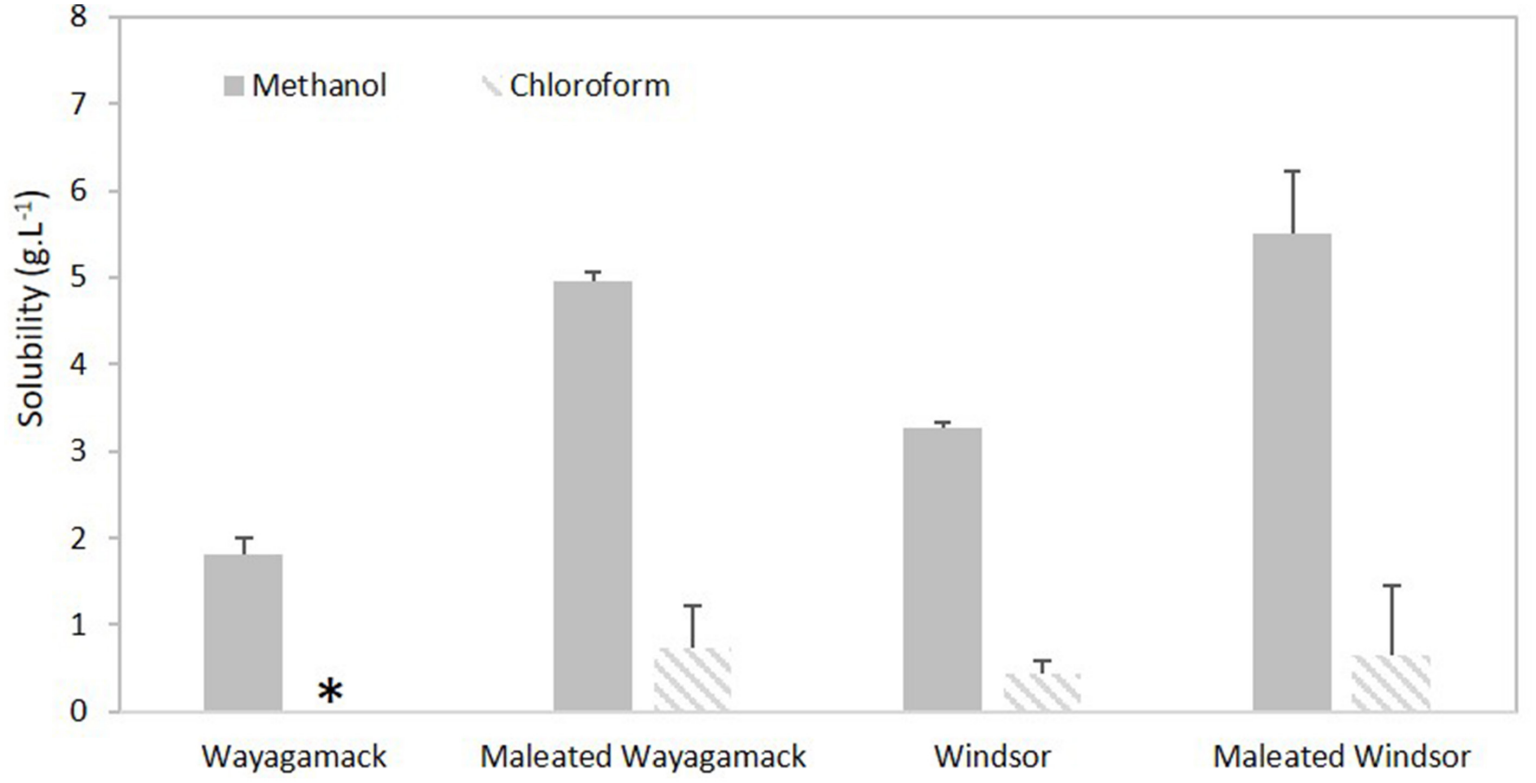
Solubility values of Wayagamack and Windsor lignins in methanol and chloroform before and after esterification. *Insoluble.

Wayagamack and Windsor lignins were selected for their contrasted H/G/S ratio (1/96/3 vs. 1/32/67, respectively). Methanol and chloroform, distinct by their solubility parameter δ from Hildebrand theory (14.3 vs. 9.2 (cal/cm^−3^)^1/2^, respectively) and hydrogen bonding parameter δ_H_ from the Hansen theory (10.9 vs. 2.8 (cal/cm^−3^)^1/2^, respectively), were chosen for this study. The solubility values of Wayagamack and Windsor lignins before and after esterification were presented in the Figure 7. Windsor lignin exhibited a higher solubility values than Wayagamack lignin in methanol (3.3 vs. 1.8 g.L^−1^) and chloroform (0.43 g.L^−1^ vs. insoluble). As expected, methanol was a more suitable solvent than chloroform to solubilize lignins according to their respective δ and δ_H_ parameters. Based on molecular weight measurements reported in Schorr et al. (2014), we suggested that the higher solubility of Windsor lignin in a given solvent in comparison to Wayagamack lignin could be explained by its lower molecular weight (Mw 3863 vs. 4859, respectively). In addition, this higher solubility could be also related to the higher HO_total_ concentration in Windsor lignin than in Wayagamack lignin (Table 3) as already suggested (Sameni et al., 2017). In addition, whatever the lignin, esterification induced an increase in solubility values in methanol (x 2.7 for Wayagamack lignin and x 1.6 for Windsor). This improvement could be related to mono-acylation by maleic anhydride leading to additional exposed carboxylic groups. Indeed, these groups would allow additional hydrogen bonding with solvent and so a better solvation of the polymer. In chloroform, this improvement was less marked with a solubility of maleated lignins inferior to 1 g.L^−1^. Nevertheless, Wayagamack lignin, initially insoluble in this solvent became slightly more soluble. This weak solubility improvement in aprotic organic solvent as chloroform seemed to be due to a compromise induced by the covalent grafting of maleic anhydride, providing both additional alkyl chains favorable to solvation in this solvent and additional free carboxylic groups which might be unfavorable.

## Conclusion

Chemical esterifications of four industrial lignins, distinct by their origin, and extraction process, were succeeded for the first time with maleic anhydride in acidic ionic liquid. This route was easy to implement, fast and did not require additional catalyst. An excess of acyl donor favored the efficiency of the reaction whatever the origin and extraction process of lignin. Between 30 to 52% of hydroxyls of lignin were esterified. For three out of four lignins (Indulin AT, Windsor, Lignol), the regioselectivity of the reaction system was mainly orientated toward aliphatic hydroxyls (>60%) reflecting both their accessibility in the molecular environment and their reactivity. For the Wayagamack lignin, esterification yield was the lowest but the absence of selectivity between aliphatic and phenolic hydroxyl suggested an improved reactivity of phenolic hydroxyls in our reaction system. Esterification of lignins with maleic anhydride increased significantly their solubility in polar and protic solvent probably due to additional exposed carboxylic groups resulted from mono-acylation. Although the covalent grafting of maleyl chains on lignin induced a very slight decrease in thermal stability, this remained compatible with temperature conditions of extrusion process for the conception of partially biosourced composites.

## Data Availability

All datasets generated for this study are included in the manuscript and the Supplementary Files.

## Author Contributions

EH, CS, TS, and LH conceived and designed the experiments. LH performed and analyzed the experiments with the help of EH, CS for esterification conditions, choices of IL, and analytical methods, and TS for choosing the raw materials and the grafting and the thermal properties analyses. CH helped for physico-chemical interpretations. EH and CS wrote the manuscript on the basis of LH Ph.D. dissertation. CB performed required experiments for the revision. All authors helped with drafting the manuscript and approved the final version.

### Conflict of Interest Statement

The authors declare that the research was conducted in the absence of any commercial or financial relationships that could be construed as a potential conflict of interest.
