# Supplementary material for: Acidic Ionic Liquid as Both Solvent and Catalyst for Fast Chemical Esterification of Industrial Lignins: Performances and Regioselectivity
Source: Front Chem. 2019 Aug 14;7:578. doi: 10.3389/fchem.2019.00578 (PMC6705185; doi:10.3389/fchem.2019.00578)
Supplement: Supplementary file 1 [file Data_Sheet_1.docx]

Supplementary Material

**Supplementary Table 1**. Chemical composition and ash content of Kraft lignins adapted from Schorr *et al.* 2014.

| **Composition (%)** | **Industrial lignins** | | |
| --- | --- | --- | --- |
|  | Indulin AT | Wayagamack | Windsor |
| Klason lignin | 92 ± 2 | 91 ± 1 | 91 ± 1 |
| Acid soluble lignin | 4.4 ± 0.3 | 5.7 ± 0.5 | 7.0 ± 2.0 |
| Total sugars | 1.0 ± 0.2 | 1.2 ± 0.1 | 0.67 ± 0.04 |
| Arabinose | nd | nd | 5 |
| Galactose | 10 | 59 | 11 |
| Glucose | 12 | 19 | 21 |
| Xylose | 5 | 6 | 60 |
| Mannose | 73 | 71 | 3 |
| Ash content | 3.59 ± 0.7 | 0.73 ± 0.08 | 0.47 ± 0.02 |

nd: not detected

**Supplementary Table 2.** Relative composition in degradation products of the four industrial lignins obtained by Pyrolysis-GC/MS results and their respective H/G/S ratios.

| **Degradation products** | **Industrial lignins** | | | |
| --- | --- | --- | --- | --- |
|  | **Indulin AT** | **Wayagamack** | **Lignol** | **Windsor** |
|  | Relative area (%) | | | |
| Hydroxyphenyl units (H) | | | | |
| Phenol | 1.1 | 0.4 | 0.6 | 0.2 |
| Cresol ortho | 0.7 | 0.6 | 0.4 | 0.2 |
| Cresol para/meta | 0.9 | 0.5 | 1 | 0.3 |
| Dimethyl phenol | 0.8 | - | 0.8 | - |
| 2-ethylphenol | 0.1 | - | - | - |
| 3-ethylphenol | - | - | 0.2 | - |
| 4-ethylphenol | 0.4 | - | - | 0.5 |
| Cuminaldehyde | - | - | 0.4 | - |
| 4-allylphenol | - | - | 0.3 | - |
| Total | 4.0 | 1.5 | 3.7 | 1.2 |
| Guaiacyl units (G) | | | | |
| Guaiacol | 22.5 | 9.2 | 8.7 | 3.9 |
| 6-methylguaiacol | - | - | 4.7 | - |
| 5-methylguaiacol | - | - | 0.5 | - |
| 4-methylguaiacol | 5.3 | 13 | 14.7 | 5.5 |
| 4-ethylguaiacol | 9.4 | 5 | 6.4 | 2.4 |
| 6-vinylguaiacol | 8.6 | 11.5 | 11.7 | 2.7 |
| Eugenol | 2.7 | 2.2 | 1.8 | 0.2 |
| 4-propylguaiacol | 1.9 | 5.4 | - | 2.8 |
| Vanilline | 9.1 | 1 | 4.7 | 1.6 |
| Isoeugenol (cis) | 3.3 | 6.5 | 3.8 | 1.9 |
| Isoeugenol (trans) | 11.8 | 5.9 | 4.9 | 0.5 |
| Acetoguaiacone | 5.8 | 12.9 | 3.4 | 1.5 |
| Methyl vanillate | 1 | - | 4.1 | - |
| Acetone guaiacol | 3.6 | 6.2 | 5.8 | 2.4 |
| Propioguaiacone | 3.4 | - | 6.1 | - |
| Homovanillic alcohol | 0.6 | 1.8 | 3.2 | - |
| Ethyl homovanillate |  | - | 2.5 | - |
| Coniferyl alcohol (cis) | 0.8 | - | - | - |
| Coniferyl alcohol | 4.1 | 1.4 | 5.4 | 0.3 |
| Homovanillic acid | - | 6.3 | - | - |
| Total | 93.9 | 88.3 | 92.4 | 25.7 |
| Syringyl units (S) | | | | |
| Syringol | 1.2 | 0.2 | 1 | 13.6 |
| 4-propenylsyringol | 0.2 | 0.2 | 0.7 | 6.1 |
| 4-allylsyringol | 0.2 | - | 0.4 | - |
| Syringaldehyde | 0.4 | 1 | 0.8 | 3.1 |
| Acetosyringone | 0.3 | - | 0.4 | 1.5 |
| 3.5-dimethoxyacetophenone | - | - | - | 6.2 |
| Cis-4-propenylsyringol | - | - | - | 1.2 |
| 4-methylsyringol | - | - | - | 14.2 |
| Other | - | 1.3 | - | 7.6 |
| Total | 2.3 | 2.7 | 3.2 | 53.5 |
| **Ratio H/G/S** | **4/94/2** | **1/96/3** | **4/93/3** | **1/32/67** |

Pyrolysis-GC/MS analyses were performed according experimental procedure described by Schorr et al. (2014). Each lignin was analyzed in triplicate. Each peak of chromatograms was identified according to NIST Mass Spectral Library and literature data (Meier et al. 1992 ; Sun, 2010). H/G/S ratio were calculated based on relative area (%) of each degradation product.

**Supplementary Table 3.** Condensation index and density of raw lignins.

|  | Indulin AT^a^ | Wayagamack^b^ | Lignol^a^ | Windsor^b^ |
| --- | --- | --- | --- | --- |
| Condensation index | 0.609 ± 0.002 | 0.618 | 0.599 ± 0.004 | 0.454 |
| density (g.cm^-3^) | 1.242 ± 0.003 | nd | 1.339 ± 0.001 | nd |

^a^ Condensation index was determined using IR spectroscopy and the following equation (Faix et al. 1991), Sum of all minima intensities between 1500 and 1050 cm^-1^ / Sum of all maxima intensities between 1600 and 1030 cm^-1^; density was determined by gas picnometer UltraPyc1200e Automatic density analyzer (Quantachrome instruments, Anton Paar, US). 500 mg of sample was required for this analysis. Each analysis was repeated in triplicate and density values were expressed as mean values with standard deviations (±). ^b^ Adapted from Schorr *et al.* 2014; nd : not determined.


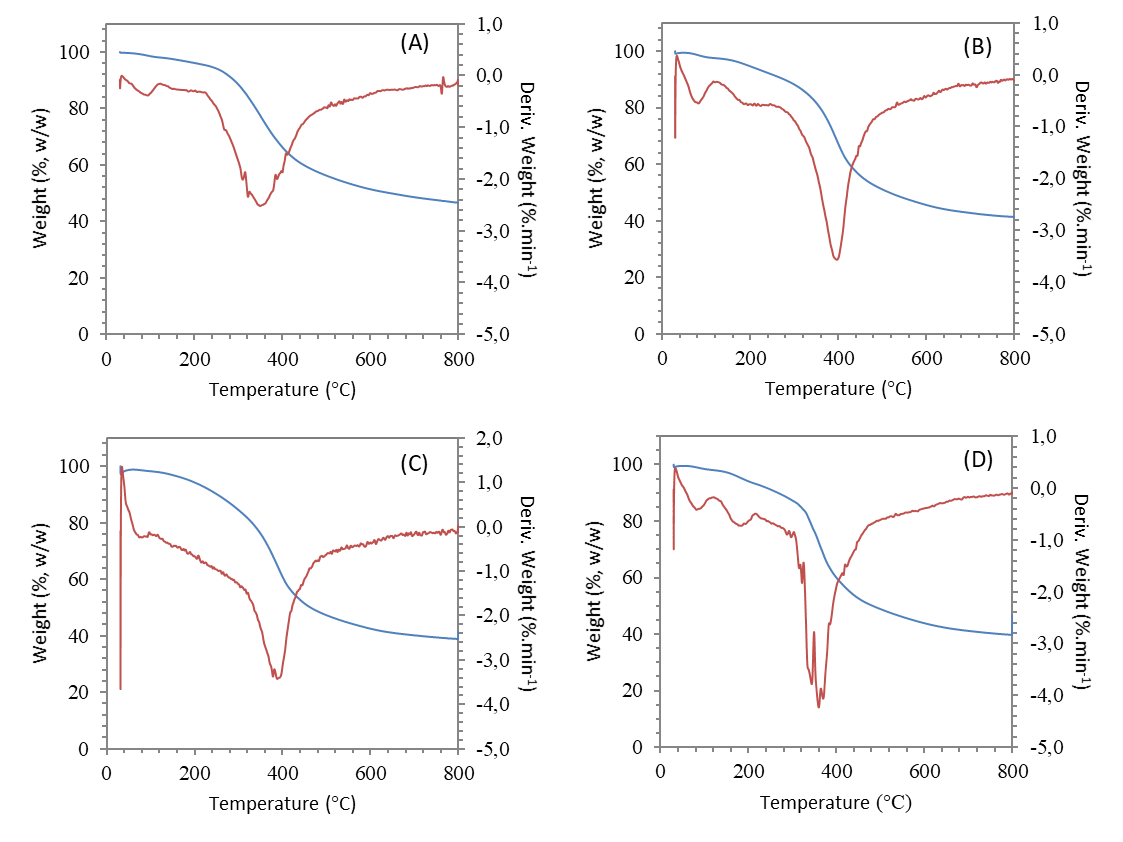


**Supplementary Figure 1**. TGA (blue curve) and DTG (red curve) thermogramms of the four raw lignins: Indulin AT lignin (A), Wayagamack lignin (B), Lignol lignin (C) and Windsor lignin (D).


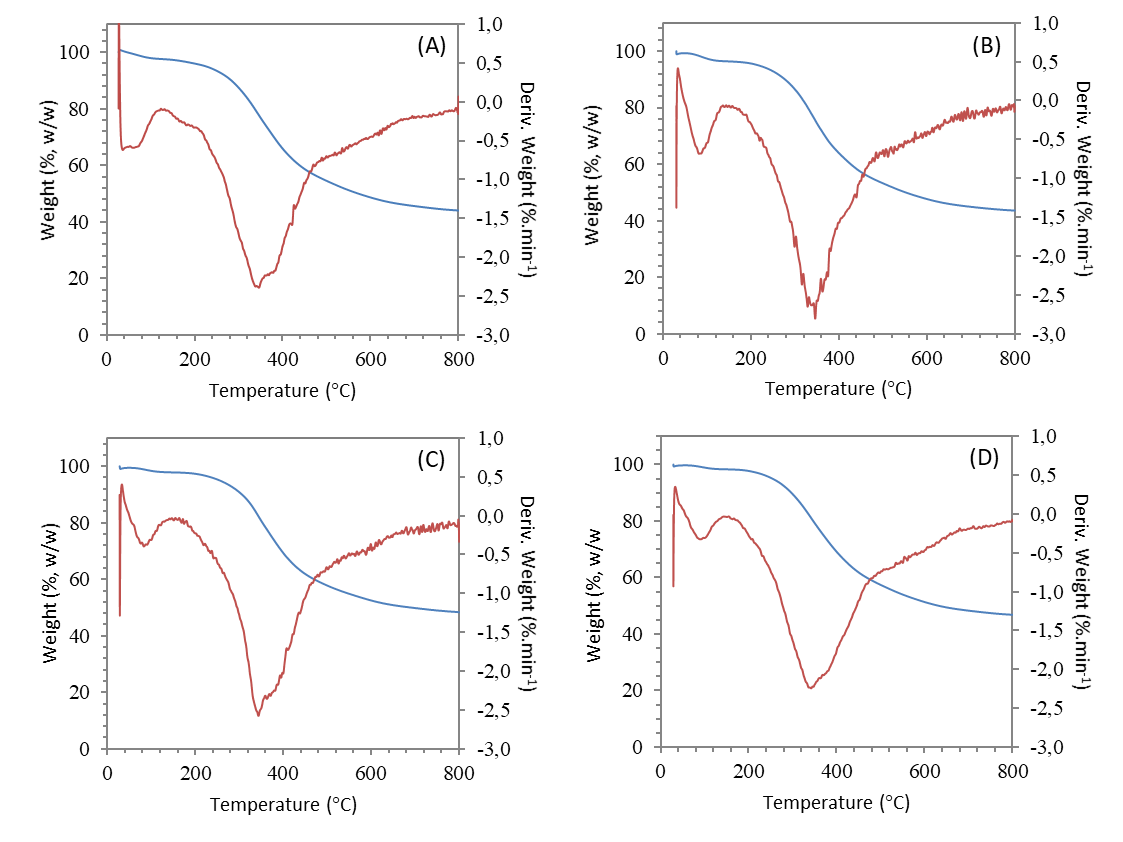


**Supplementary Figure 2**. TGA (blue curve) and DTG (red curve) thermogramms of the four lignin controls: Indulin AT lignin (A), Wayagamack lignin (B), Lignol lignin (C) and Windsor lignin (D) previously incubated in [Bmim][HSO_4_] without maleic anhydride.


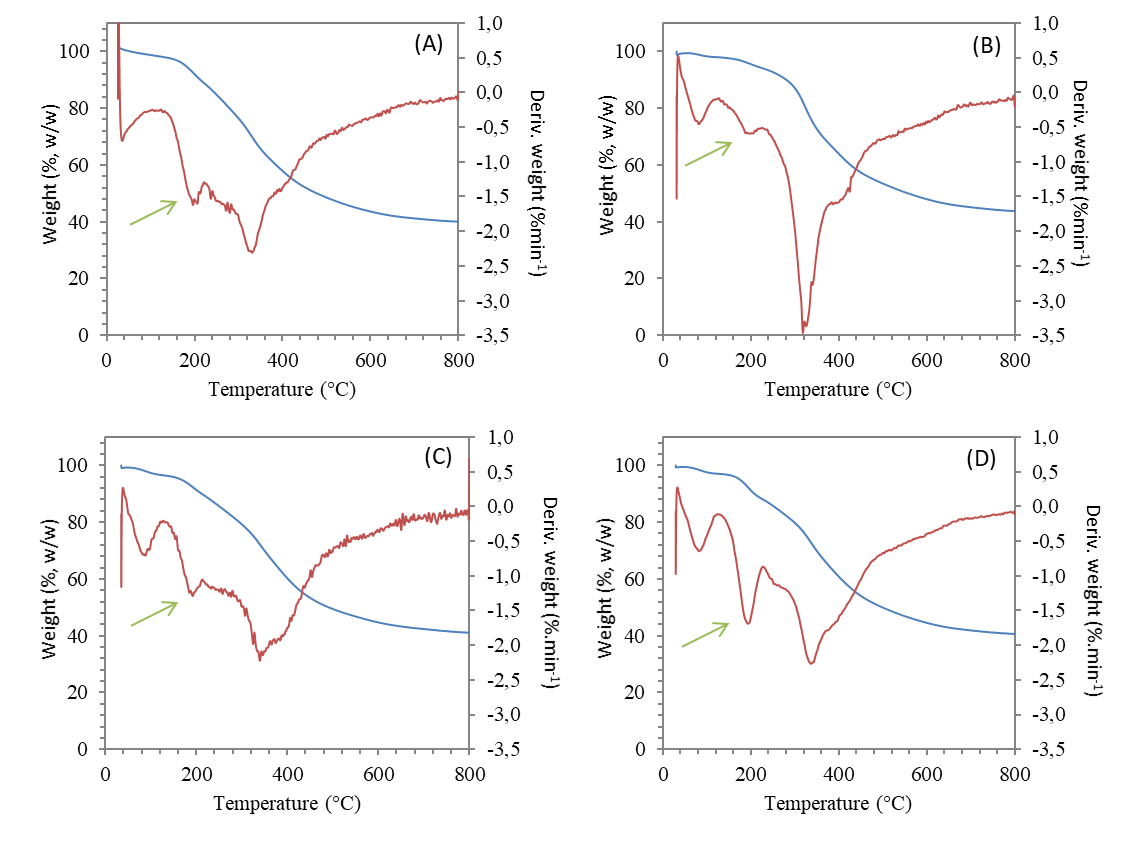


**Supplementary Figure 3**. TGA (blue curve) and DTG (red curve) thermogramms of maleated Indulin AT lignin (A), maleated Wayagamack lignin (B), maleated Lignol lignin (C) and maleated Windsor lignin (D) synthesized in [Bmim][HSO_4_] with maleic anhydride.


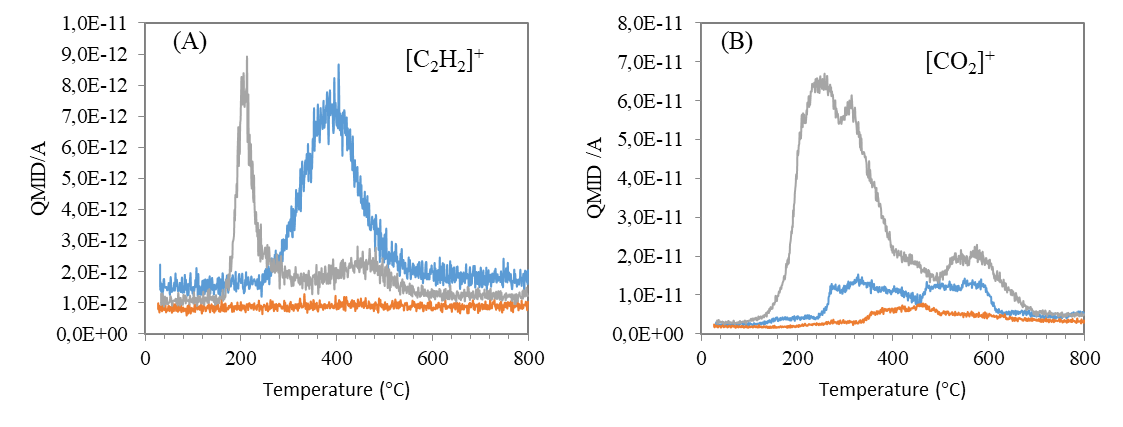


**Supplementary Figure 4.** TGA-MS coupled analysis of raw Indulin AT (blue), control Indulin AT (red) and Indulin AT maleate (green) as function of temperature. Ionic current for m/z = 26 (A) and 44 (B).
